# Special Issue: Bioactive Compounds, Nutritional Quality, and Oxidative Stability of Edible Oils and By-Products of Their Extraction

**DOI:** 10.3390/foods12163133

**Published:** 2023-08-21

**Authors:** Katarzyna Ratusz, Małgorzata Wroniak, Iwona Ścibisz

**Affiliations:** 1 Division of Fats and Oils Technology, Department of Food Technology and Assessment, Institute of Food Sciences, Warsaw University of Life Sciences, Nowoursynowska 159c, 02-776 Warsaw, Poland; 2 Division of Fruit, Vegetable and Cereal Technology, Department of Food Technology and Assessment, Institute of Food Sciences, Warsaw University of Life Sciences, Nowoursynowska 159c, 02-776 Warsaw, Poland; iwona_scibisz@sggw.edu.pl

## 1. Introduction

Edible oils (refined, virgin, and cold-pressed oils) are one of the most important components of the daily human diet and have a considerable influence on the proper functioning of our body. However, their nutritional quality varies greatly. Many factors affect the security and quality of edible oils. The most important is, of course, the raw material from which the oil was obtained (the composition of fatty acids, and the content of bioactive ingredients). The methods of extracting and purifying the oils and the parameters of these processes also have a significant impact. Unfortunately, the undesirable hydrolytic and oxidative degradation processes of fats can be initiated during their production. Hence, it is imperative to closely observe shifts in oil quality throughout the technological process and take steps to mitigate these adverse changes. Similarly, the storage conditions of oils are of significant importance [1,2].

It is with great pleasure that we present this Special Issue, which focuses on analyzing the physicochemical and nutritional quality of edible oils and by-products of their extraction and on analyzing the impact of various factors on the quality and safety of oils.

## 2. Factors Affecting the Quality and Safety of Edible Oils and Their By-Products

The collection of papers included in this Special Issue offers valuable insights into the diverse range of research in this field. Several articles present the characteristics, chemical composition, oxidative stability, nutritional quality, and effects of oilseeds and oils on human health. One of the articles here [3] discusses the characteristics and effects of black seed (*Nigella sativa*) on human health. Black seed and its oil contain thymoquinone, which has antioxidant, antidiabetic, anti-inflammatory, anticancer, antiviral, and antimicrobial properties due to its phenolic compounds. Various methods of extraction and of increasing stability using different encapsulation techniques (including nanoprecipitation, ultrasonication, spray-drying, electrohydrodynamic atomization, freeze-drying, electrospray technique, and coaxial electrospraying) are also presented. Another article [4] discusses *Idesia polycarpa* pulp oil. This potentially high-quality vegetable oil was studied for its chemical parameters, fatty acid composition, bioactive ingredients, and antioxidant capacity across different Chinese regions. Variations were observed among regions, influencing the oil’s nutritional and industrial applications.

Oilseeds and some of their by-products have raised interest in the food and pharmaceutical industries, seeking innovative products whose applications provide health benefits to consumers. One of the articles here [5] describes different oilseeds as sources of bioactive compounds with health benefits, promoting cardiovascular health and preventing diseases. Additionally, different types of extraction techniques that can be used to obtain vegetable oils rich in oilseeds, such as microwave-assisted extraction, ultrasonic-assisted extraction, and supercritical fluid extraction, are outlined. One of the most important characteristics of fats and oils is their oxidative stability. In one of the articles presented here [6], selected cold-pressed oils (pumpkin, hemp, linseed, camelina, rapeseed, black cumin, milk thistle, evening primrose, and several blends of different cold-pressed oils) were analyzed for their chemical composition, total phenolic compounds, DPPH and ABTS free radical scavenging activity, and oxidative stability (Rancimat test). The study concluded that various factors influenced the stability of the oils, and correlations between different properties were observed.

The results presented in another article were also very interesting. The effect of gelatin strips with polyvinyl alcohol and sinapic acid esters (ethyl sinapate, octyl sinapate, and cetyl sinapate) on the oxidative stability, antioxidant activity, and total phenolic content in cold-pressed rapeseed oil samples was analyzed during accelerated storage [1]. This study revealed that gelatin strips with polyvinyl alcohol incorporated with sinapic acid esters enhanced the antioxidant potential of oil and delayed oxidative degradation by releasing amphiphilic antioxidants into the oil.

In another study [2], the influence of mullein flower extract addition on the oxidative stability and antioxidant activity of cold-pressed oils with a high content of unsaturated fatty acids (rapeseed, chia seed, linseed, and hempseed) was examined. The conducted research showed that the addition of mullein flower extract increases the oxidative stability of oils, but the optimal amount of its addition depends on the type of oil and should be determined through experimental selection.

In another article [7], the effects of ferulic acid and its derivatives on the oxidative stability of cold-pressed flaxseed oil were investigated. These compounds influenced the oxidative stability and degradation of bioactive components, with concentration and temperature playing significant roles. In another work [8], the composition and oxidative stability of cold-pressed hemp seed oils from stored and fresh seeds were analyzed. While differences were observed in some properties, the oxidative stability did not show significant variation between the two groups.

Many articles in this Special Issue analyze the impact of technological processes on the quality of oils. For example, one study [9] investigated the effects of seed age and clarification processes on the oxidative stability and phase transition of raspberry seed oil. Centrifugation of the oil lowered the quality parameters, which suggests that oil clarification processes should not be abandoned. Another article [10] compared different processing techniques of camellia oil in terms of triacylglycerol profile, bioactive compounds, oxidative stability, and volatile compounds. The fresh pressing technique showed a higher content of bioactive compounds and greater antioxidant capacity.

Another article [11] investigated the effect of the refining process on the quality and stability of fractionated and mixed shea butter. Crude shea butter, refined shea stearin, olein, and their mixture were analyzed for fatty acids, triacylglycerol composition, phenolic flavonoid, unsaponifiable matter, tocopherol content, and phytosterol content. The oxidative stability, radical scavenging activity, and antibacterial and antifungal activities were also evaluated. 

It was also interesting to explore the influence of the method of extracting oil from sinami fruit (*Oenocarpus mapora* H. Karst) on its quality [12]. The physicochemical properties, total polyphenol content, and antioxidant activity of sinami oil that was obtained using four extraction systems—namely expeller-press extraction, cold-press extraction, ultrasound-assisted extraction and supercritical fluid extraction—were studied and compared.

Another article [13] reported the characteristics of new oil blends with a nutritious ω6/ω3 fatty acid ratio (5:1), as well as the effect of heat treatment on the nutritional value and stability of the oils.

The technological process parameters also influence the quality of by-products such as press cake obtained during the pressing and extraction of meal. Presently, there is an increasing focus on zero-waste technology and the management of by-products generated from the processing of oil raw materials. There is extensive research being conducted to create new and innovative food products that utilize the by-products of vegetable oil pressing. In this Special Issue, the results of work on the development of a nutraceutical product in bar form using defatted Brazil nut by-products are presented [14].

## 3. Concluding Remarks

In conclusion, this Special Issue brings together a collection of research papers that highlight the impact of the quality of raw material, processing techniques, and extraction methods on the composition, stability, and potential health benefits of different oils. We hope that the papers in this Special Issue provide valuable insights and inspire further research on improving the quality and safety of edible oils.

## Data Availability

The data that support the findings of this study are the articles published in the aforementioned Special Issue. These data were derived from the following resources available in: https://www.mdpi.com/journal/foods/special_issues/Bioactive_Compounds_Nutritional_Quality_Oxidative_Stability_Edible_Oils_By_Products_Their_Extraction.
